# Crystal structure of tetra­aqua­bis­(pyrimidin-1-ium-4,6-diolato-κ*O* ^4^)manganese(II)

**DOI:** 10.1107/S2056989017004649

**Published:** 2017-03-31

**Authors:** Khaled A. Shennara, Ray J. Butcher, Frederick T. Greenaway

**Affiliations:** a Carlson School of Chemistry and Biochemistry, Clark University, 950 Main St, Worcester, MA 01610, USA; b Department of Chemistry, Howard University, Washington, DC 20059, USA

**Keywords:** crystal structure, 4,6-di­hydroxy­pyrimidine, manganese(II), octa­hedral coordination

## Abstract

The Mn^II^ ion in the structure of the mononuclear title compound, [Mn(C_4_H_3_N_2_O_2_)_2_(H_2_O)_4_], is situated on an inversion center and is coordinated by two O atoms from two deprotonated 4,6-di­hydroxy­pyrimidine ligands and by four O atoms from water mol­ecules giving rise to a slightly distorted octa­hedral coordination sphere. The complex includes an intra­molecular hydrogen bond between an aqua ligand and the non-protonated N ring atom. The extended structure is stabilized by inter­molecular hydrogen bonds between aqua ligands, by hydrogen bonds between N and O atoms of the ligands of adjacent mol­ecules, and by hydrogen bonds between aqua ligands and the non-coordinating O atom of an adjacent mol­ecule.

## Abstract 2

The crystal structure of the Mn^II^ complex of 4,6-di­hydroxy­primidine (*L*), [Mn*L*_2_(H_2_O)_4_], shows that the ligand coordinates to the metal ion through one deprotonated hy­droxy group from each of two ligands.

## Chemical context   

H-tautomeric forms of 4,6-di­hydroxy­pyrimidine (DHP) are known to exist and are associated with low disproportionation energies (Katrusiak & Katrusiak, 2003 ▸). Although crystal structures have been reported where cobalt(II) and nickel(II) are coordinated by the 4,6-di­hydroxy­pyrimidine ligand through a ring nitro­gen atom (Huang *et al.*, 2005 ▸; Wang *et al.*, 2006 ▸), prior to this report no complexes with ligation through a phenolate oxygen atom have been reported even though this mode of coordination does occur in complexes of 3,6-di­hydroxy­pyridizine (Shennara *et al.*, 2015 ▸).
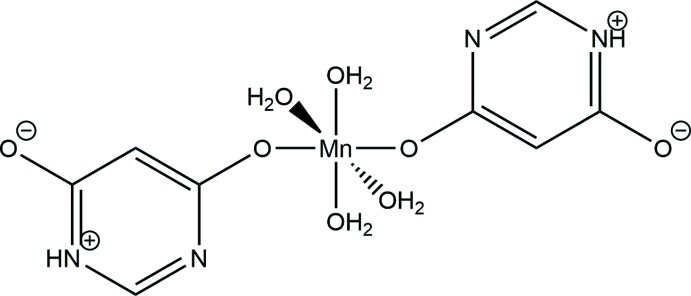


## Structural commentary   

**Figure 1:**
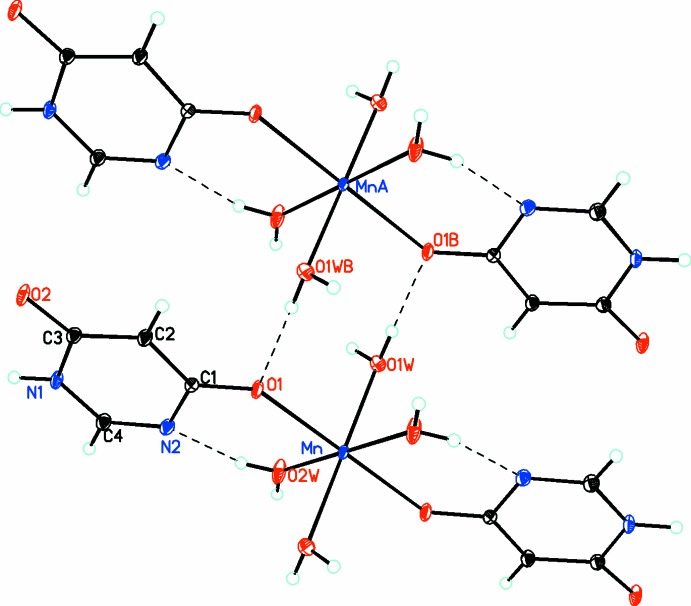
Diagram showing the complex and atom labeling, as well as the formation of {*C*(4)[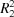
(8)]} chains in the *a*-axis direction linked by hydrogen bonds. Atomic displacement parameters are drawn at the 50% probability level. Hydrogen bonds are shown as dashed lines.

Crystallographic analysis reveals that the title compound consists of a centrosymmetric mononuclear [Mn(C_4_H_3_N_2_O_2_)_2_(H_2_O)_4_] complex in which the Mn^II^ ion is in an O_6_ environment that is close to octa­hedral. Two deprotonated 4,6-di­hydroxy­pyrimidine ligands coordinate through the phenolate oxygen atom (O1) at axial positions, while four water mol­ecules occupy the equatorial sites (Fig. 1 ▸). The bond lengths in the pyrimidine ligand are very similar to those found for the Co and Ni complexes in which, however, ligation to the metal is through a nitro­gen atom. For all three complexes, the structures indicate a zwitterionic form of the ligand resulting from transfer of a proton from the hydroxyl group to a ring nitro­gen atom. Others have reported variability in the H-tautomeric forms of 4,6-di­hydroxy­pyrimidine associated with low disproportionation energies (Katrusiak & Katrusiak, 2003 ▸). The structure of the complex includes an intra­molecular hydrogen bond between an aqua ligand (O2*W*) and the non-protonated N^3^ ring atom (N2) (Table 1 ▸).

## Supra­molecular features   

**Figure 2:**
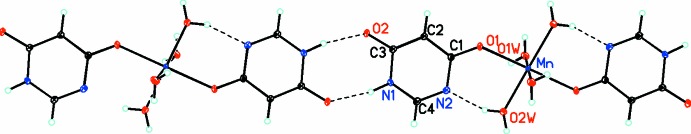
Diagram showing how the mol­ecules link up into chains through the formation of *C*(6)[*R*(6)
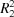
(8)] hydrogen bonds. Atomic displacement parameters are drawn at the 50% probability level. Hydrogen bonds are shown as dashed lines.

**Figure 3:**
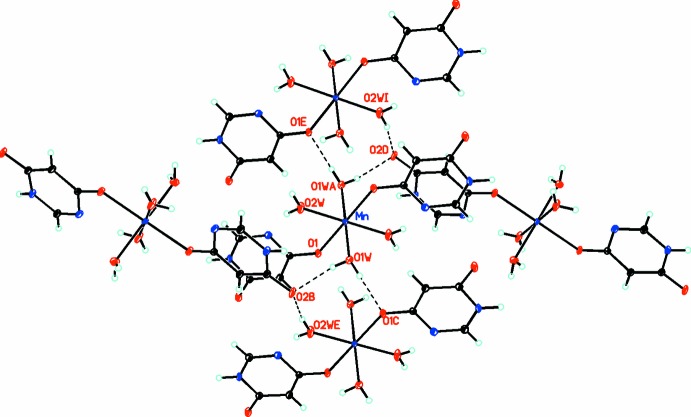
Diagram showing one of the two mutually perpendicular chains linked through the formation of *C*(6)[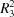
(8)] hydrogen bonds. Atomic displacement parameters are drawn at the 50% probability level. Hydrogen bonds are shown as dashed lines.

**Figure 4:**
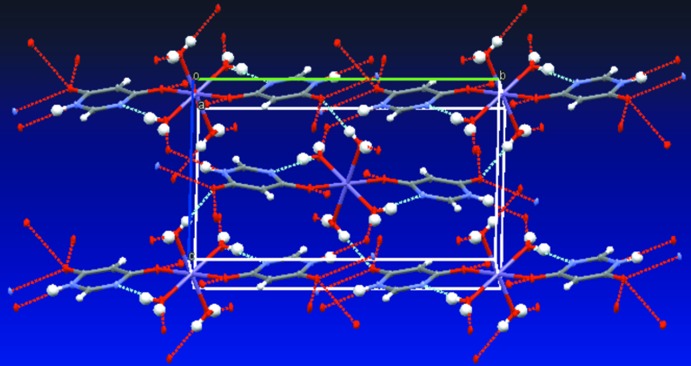
Diagram showing how the four sets of chains linked by hydrogen bonds gives rise to the overall packing. Hydrogen bonds are shown as dashed lines.

Inter­molecular hydrogen bonds between the aqua ligands of adjacent mol­ecules are present. Hydrogen bonds also occur between the non-coordinating NH^+^ and O^−^ atoms of two DHP ligands in adjacent mol­ecules and between an aqua ligand and the non-coordinating oxygen atom of an adjacent mol­ecule (Table 1 ▸). This gives rise to a complex three-dimensional network, which is best analyzed in terms of graph-set theory (Etter *et al.*, 1990 ▸). There are four inter­penetrating chains of hydrogen bonds. The first has a *C*(4)[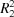
(8)] motif and is shown in Fig. 1 ▸. The second has a *C*(6)[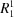
(6)
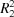
(8)] motif and is shown in Fig. 2 ▸. The chain depicted in Fig. 3 ▸ has a *C*(6)[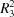
(8)] motif and is duplicated in two mutually perpendicular directions, thus making up four chains altogether. The overall packing is shown in Fig. 4 ▸.

## Database survey   

A search in the Cambridge Structural Database (CSD version 5.37; Groom *et al.*, 2016 ▸) for structures of manganese of 4,6-di­hydroxy­pyrimidines revealed that no such structures exist, although there are twelve examples of manganese complexes of 2,4-di­hydroxy­pyrimidine derivatives (CSD codes AMPTMN, AQAPAK, ICESEQ, IMEGAJ, JIRNUU, NOPSER, OFUDAU, QOSDOT, QOSNOD, RAGLAO, TAGVOM, and ZOGFOQ).

## Synthesis and crystallization   

0.5 m*M* aqueous solutions of the ligand and anhydrous MnCl_2_, both purchased from Aldrich, were adjusted to pH 5.5 with NaOH/HCl and then mixed together in a 1:2 stoichiometry. The solutions were left to crystallize slowly at room temperature. Light-yellow crystals formed over two weeks. Room-temperature X-band EPR spectra of powdered crystals exhibited a single broad line centered at a *g*-value of near to 2.0 with a peak-to-peak line width of 660 G, the breadth of which indicates Mn⋯Mn magnetic inter­actions, although not as strong as in the related maleic hydrazide (MH), Mn(MH)_2_(H_2_O)_4_, complex, for which a line width of 920 G was found (Shennara *et al.*, 2015 ▸). EPR spectra of aqueous solutions of the title complex had *g* = 2.006 and *A*_iso_(Mn) = 95.2 G, similar to that of the Mn(MH)_2_ complex

## Refinement   

Crystal data, data collection and structure refinement details are summarized in Table 2 ▸. All H atoms were positioned geometrically and refined as riding: C—H = 0.95 Å with *U*_iso_(H) = 1.2*U*_eq_(C). N—H and O—H hydrogen atoms were refined isotropically without restrictions on the bond lengths. Four reflections which were obvious outliers were omitted from the refinement (132, 163, 100, 011).

## Supplementary Material

Crystal structure: contains datablock(s) I. DOI: 10.1107/S2056989017004649/wm5373sup1.cif

Structure factors: contains datablock(s) I. DOI: 10.1107/S2056989017004649/wm5373Isup2.hkl

CCDC reference: 1539878

Additional supporting information:  crystallographic information; 3D view; checkCIF report

## Figures and Tables

**Table 1:** Hydrogen-bond geometry (Å, °)

*D*—H⋯*A*	*D*—H	H⋯*A*	*D*⋯*A*	*D*—H⋯*A*
O1*W*—H1*W*1⋯O2^i^	0.80 (3)	2.03 (3)	2.8152 (14)	170 (2)
O1*W*—H1*W*2⋯O1^ii^	0.82 (3)	1.90 (3)	2.7127 (13)	176 (3)
O2*W*—H2*W*1⋯N2	0.82 (3)	1.91 (3)	2.6929 (14)	159 (2)
O2*W*—H2*W*2⋯O2^iii^	0.84 (2)	1.85 (2)	2.6754 (13)	167 (2)
N1—H1*N*⋯O2^iv^	0.91 (2)	1.92 (2)	2.7966 (14)	162 (2)

Symmetry codes: (i) 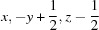; (ii) 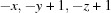; (iii) 
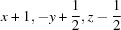
; (iv) 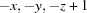.

**Table 2:** Experimental details

Crystal data	
Chemical formula	[Mn(C_4_H_3_N_2_O_2_)_2_(H_2_O)_4_]
*M*_r_	349.17
Crystal system, space group	Monoclinic, *P*2_1_/*c*
Temperature (K)	120
*a*, *b*, *c* (Å)	5.2156 (5), 14.0812 (14), 9.0595 (9)
β (°)	99.366 (2)
*V* (Å^3^)	656.48 (11)
*Z*	2
Radiation type	Mo *K*α
μ (mm^−1^)	1.05
Crystal size (mm)	0.55 × 0.41 × 0.40

Data collection	
Diffractometer	Bruker APEXII CCD
Absorption correction	Multi-scan (*SADABS*; Sheldrick, 1996 ▸)
*T*_min_, *T*_max_	0.614, 0.746
No. of measured, independent and observed [*I* > 2σ(*I*)] reflections	2971, 1848, 1752
*R*_int_	0.016
(sin θ/λ)_max_ (Å^−1^)	0.730

Refinement	
*R*[*F* ^2^ > 2σ(*F* ^2^)], *wR*(*F* ^2^), *S*	0.027, 0.071, 1.10
No. of reflections	1848
No. of parameters	117
H-atom treatment	H atoms treated by a mixture of independent and constrained refinement
Δρ_max_, Δρ_min_ (e Å^−3^)	0.48, −0.32

Computer programs: *APEX2* (Bruker, 2005 ▸), *SAINT* (Bruker, 2002 ▸), *SHELXT* (Sheldrick, 2015*a* ▸), *SHELXL2014* (Sheldrick, 2015*b* ▸) and *SHELXTL* (Sheldrick, 2008 ▸).

## supplementary crystallographic information

### Crystal data

**Table:**

[Mn(C_4_H_3_N_2_O_2_)_2_(H_2_O)_4_]	*F*(000) = 358
*M**_r_* = 349.17	*D*_x_ = 1.766 Mg m^−^^3^
Monoclinic, *P*2_1_/*c*	Mo *K*α radiation, λ = 0.71073 Å
*a* = 5.2156 (5) Å	Cell parameters from 2512 reflections
*b* = 14.0812 (14) Å	θ = 2.7–31.2°
*c* = 9.0595 (9) Å	µ = 1.05 mm^−^^1^
β = 99.366 (2)°	*T* = 120 K
*V* = 656.48 (11) Å^3^	Block, yellow
*Z* = 2	0.55 × 0.41 × 0.40 mm

### Data collection

**Table:**

Bruker APEXII CCD diffractometer	1752 reflections with *I* > 2σ(*I*)
ω scans	*R*_int_ = 0.016
Absorption correction: multi-scan (*SADABS*; Sheldrick, 1996)	θ_max_ = 31.2°, θ_min_ = 2.9°
*T*_min_ = 0.614, *T*_max_ = 0.746	*h* = −7→7
2971 measured reflections	*k* = −18→18
1848 independent reflections	*l* = −3→12

### Refinement

**Table:**

Refinement on *F*^2^	0 restraints
Least-squares matrix: full	Hydrogen site location: mixed
*R*[*F*^2^ > 2σ(*F*^2^)] = 0.027	H atoms treated by a mixture of independent and constrained refinement
*wR*(*F*^2^) = 0.071	*w* = 1/[σ^2^(*F*_o_^2^) + (0.033*P*)^2^ + 0.3858*P*] where *P* = (*F*_o_^2^ + 2*F*_c_^2^)/3
*S* = 1.10	(Δ/σ)_max_ < 0.001
1848 reflections	Δρ_max_ = 0.48 e Å^−^^3^
117 parameters	Δρ_min_ = −0.32 e Å^−^^3^

### Special details

**Table:**

Geometry. All esds (except the esd in the dihedral angle between two l.s. planes) are estimated using the full covariance matrix. The cell esds are taken into account individually in the estimation of esds in distances, angles and torsion angles; correlations between esds in cell parameters are only used when they are defined by crystal symmetry. An approximate (isotropic) treatment of cell esds is used for estimating esds involving l.s. planes.

### Fractional atomic coordinates and isotropic or equivalent isotropic displacement parameters (Å^2^)

**Table:**

	*x*	*y*	*z*	*U*_iso_*/*U*_eq_	
Mn	0.500000	0.500000	0.500000	0.00759 (9)	
O1	0.25688 (17)	0.38379 (6)	0.56407 (10)	0.01144 (18)	
O2	−0.08946 (18)	0.07807 (6)	0.62265 (10)	0.01204 (18)	
O1W	0.17104 (18)	0.54921 (7)	0.33556 (11)	0.01243 (18)	
H1W1	0.114 (5)	0.5129 (16)	0.271 (3)	0.029 (6)*	
H1W2	0.045 (5)	0.5682 (17)	0.370 (3)	0.040 (7)*	
O2W	0.6010 (2)	0.40720 (7)	0.32982 (11)	0.0164 (2)	
H2W1	0.562 (5)	0.3534 (18)	0.353 (3)	0.037 (6)*	
H2W2	0.702 (5)	0.4026 (17)	0.267 (3)	0.035 (6)*	
N1	0.2263 (2)	0.10486 (8)	0.48187 (11)	0.0094 (2)	
H1N	0.219 (5)	0.0429 (18)	0.453 (3)	0.033 (6)*	
N2	0.4089 (2)	0.25411 (8)	0.45266 (11)	0.0102 (2)	
C1	0.2421 (2)	0.29415 (9)	0.54070 (13)	0.0084 (2)	
C2	0.0651 (2)	0.23594 (9)	0.60057 (13)	0.0101 (2)	
H2A	−0.049639	0.263207	0.660180	0.012*	
C3	0.0573 (2)	0.13885 (9)	0.57302 (13)	0.0090 (2)	
C4	0.3920 (2)	0.16342 (9)	0.42645 (13)	0.0102 (2)	
H4A	0.503298	0.136653	0.364320	0.012*	

### Atomic displacement parameters (Å^2^)

**Table:**

	*U*^11^	*U*^22^	*U*^33^	*U*^12^	*U*^13^	*U*^23^
Mn	0.00731 (13)	0.00453 (14)	0.01196 (13)	−0.00112 (8)	0.00470 (9)	−0.00066 (8)
O1	0.0118 (4)	0.0049 (4)	0.0192 (4)	−0.0018 (3)	0.0074 (3)	−0.0011 (3)
O2	0.0165 (4)	0.0057 (4)	0.0163 (4)	−0.0030 (3)	0.0097 (3)	−0.0001 (3)
O1W	0.0105 (4)	0.0099 (5)	0.0173 (4)	0.0014 (3)	0.0032 (3)	−0.0025 (3)
O2W	0.0242 (5)	0.0076 (5)	0.0219 (5)	−0.0037 (4)	0.0172 (4)	−0.0024 (3)
N1	0.0123 (5)	0.0051 (5)	0.0121 (4)	−0.0013 (3)	0.0058 (4)	−0.0019 (3)
N2	0.0096 (5)	0.0085 (5)	0.0134 (4)	−0.0014 (3)	0.0050 (4)	−0.0002 (4)
C1	0.0073 (5)	0.0070 (5)	0.0110 (4)	−0.0006 (4)	0.0018 (4)	0.0000 (4)
C2	0.0101 (5)	0.0074 (6)	0.0143 (5)	−0.0010 (4)	0.0063 (4)	−0.0002 (4)
C3	0.0091 (5)	0.0082 (6)	0.0106 (4)	−0.0007 (4)	0.0040 (4)	0.0002 (4)
C4	0.0108 (5)	0.0090 (6)	0.0118 (5)	−0.0009 (4)	0.0049 (4)	0.0007 (4)

### Geometric parameters (Å, º)

**Table:**

Mn—O2W	2.1510 (10)	O2W—H2W2	0.84 (2)
Mn—O2W^i^	2.1510 (10)	N1—C4	1.3491 (15)
Mn—O1W	2.1934 (10)	N1—C3	1.3871 (14)
Mn—O1W^i^	2.1935 (10)	N1—H1N	0.91 (2)
Mn—O1	2.2050 (9)	N2—C4	1.2993 (16)
Mn—O1^i^	2.2050 (9)	N2—C1	1.3917 (15)
O1—C1	1.2801 (15)	C1—C2	1.4074 (16)
O2—C3	1.2778 (14)	C2—C3	1.3892 (17)
O1W—H1W1	0.80 (3)	C2—H2A	0.9500
O1W—H1W2	0.82 (3)	C4—H4A	0.9500
O2W—H2W1	0.82 (3)		
			
O2W—Mn—O2W^i^	180.0	Mn—O2W—H2W1	106.3 (17)
O2W—Mn—O1W	87.76 (4)	Mn—O2W—H2W2	141.9 (16)
O2W^i^—Mn—O1W	92.24 (4)	H2W1—O2W—H2W2	108 (2)
O2W—Mn—O1W^i^	92.25 (4)	C4—N1—C3	121.20 (10)
O2W^i^—Mn—O1W^i^	87.75 (4)	C4—N1—H1N	118.5 (14)
O1W—Mn—O1W^i^	180.0	C3—N1—H1N	120.1 (15)
O2W—Mn—O1	87.49 (4)	C4—N2—C1	118.21 (10)
O2W^i^—Mn—O1	92.51 (4)	O1—C1—N2	117.88 (10)
O1W—Mn—O1	89.62 (4)	O1—C1—C2	122.43 (11)
O1W^i^—Mn—O1	90.38 (4)	N2—C1—C2	119.69 (11)
O2W—Mn—O1^i^	92.51 (4)	C3—C2—C1	120.38 (11)
O2W^i^—Mn—O1^i^	87.49 (4)	C3—C2—H2A	119.8
O1W—Mn—O1^i^	90.38 (4)	C1—C2—H2A	119.8
O1W^i^—Mn—O1^i^	89.62 (4)	O2—C3—N1	116.96 (11)
O1—Mn—O1^i^	180.0	O2—C3—C2	126.70 (11)
C1—O1—Mn	135.24 (8)	N1—C3—C2	116.34 (10)
Mn—O1W—H1W1	116.9 (17)	N2—C4—N1	124.15 (11)
Mn—O1W—H1W2	115.8 (18)	N2—C4—H4A	117.9
H1W1—O1W—H1W2	105 (2)	N1—C4—H4A	117.9
			
Mn—O1—C1—N2	−1.40 (18)	C4—N1—C3—O2	−178.77 (11)
Mn—O1—C1—C2	178.55 (9)	C4—N1—C3—C2	1.38 (17)
C4—N2—C1—O1	−178.88 (11)	C1—C2—C3—O2	178.56 (12)
C4—N2—C1—C2	1.17 (17)	C1—C2—C3—N1	−1.61 (17)
O1—C1—C2—C3	−179.55 (11)	C1—N2—C4—N1	−1.49 (18)
N2—C1—C2—C3	0.39 (18)	C3—N1—C4—N2	0.19 (19)

Symmetry code: (i) −*x*+1, −*y*+1, −*z*+1.

### Hydrogen-bond geometry (Å, º)

**Table:**

*D*—H···*A*	*D*—H	H···*A*	*D*···*A*	*D*—H···*A*
O1*W*—H1*W*1···O2^ii^	0.80 (3)	2.03 (3)	2.8152 (14)	170 (2)
O1*W*—H1*W*2···O1^iii^	0.82 (3)	1.90 (3)	2.7127 (13)	176 (3)
O2*W*—H2*W*1···N2	0.82 (3)	1.91 (3)	2.6929 (14)	159 (2)
O2*W*—H2*W*2···O2^iv^	0.84 (2)	1.85 (2)	2.6754 (13)	167 (2)
N1—H1*N*···O2^v^	0.91 (2)	1.92 (2)	2.7966 (14)	162 (2)

Symmetry codes: (ii) *x*, −*y*+1/2, *z*−1/2; (iii) −*x*, −*y*+1, −*z*+1; (iv) *x*+1, −*y*+1/2, *z*−1/2; (v) −*x*, −*y*, −*z*+1.

## References

[bb1] Bruker (2002). *SAINT*. Bruker AXS Inc., Madison, Wisconsin, USA.

[bb2] Bruker (2005). *APEX2*. Bruker AXS Inc., Madison, Wisconsin, USA.

[bb3] Etter, M. C., MacDonald, J. C. & Bernstein, J. (1990). *Acta Cryst.* B**46**, 256–262. 10.1107/s0108768189012929 2344397

[bb4] Groom, C. R., Bruno, I. J., Lightfoot, M. P. & Ward, S. C. (2016). *Acta Cryst.* B**72**, 171–179. 10.1107/S2052520616003954 PMC4822653 27048719

[bb5] Huang, Y.-G., Zhou, Y.-F., Yuan, D.-Q., Wu, B.-L. & Hong, M.-C. (2005). *Acta Cryst.* E**61**, m832–m834.

[bb6] Katrusiak, A. & Katrusiak, A. (2003). *Org. Lett.* **5**, 1903–1905. 10.1021/ol034494r 12762682

[bb7] Sheldrick, G. M. (1996). *SADABS*. University of Göttingen, Germany.

[bb8] Sheldrick, G. M. (2008). *Acta Cryst.* A**64**, 112–122. 10.1107/S0108767307043930 18156677

[bb9] Sheldrick, G. M. (2015*a*). *Acta Cryst.* A**71**, 3–8.

[bb10] Sheldrick, G. M. (2015*b*). *Acta Cryst.* C**71**, 3–8.

[bb11] Shennara, K. A., Butcher, R. J. & Greenaway, F. T. (2015). *Inorg. Chim. Acta*, **425**, 247–254.

[bb12] Wang, Y.-T., Lou, X.-H., Wang, J.-G. & Fan, Y.-T. (2006). *Acta Cryst.* E**62**, m1924–m1926.

